# Multiple object-tracking isolates feedback-specific load in attention and learning

**DOI:** 10.1167/jov.20.5.9

**Published:** 2020-05-22

**Authors:** Domenico Tullo, Chiara Perico, Jocelyn Faubert, Armando Bertone

**Affiliations:** 1 Department of Education & Counselling Psychology, McGill University, Montréal, Québec, Canada; 2 Department of Education & Counselling Psychology, McGill University, Montréal, Québec, Canada; 3 École d'optométrie, Université de Montréal, Montréal, Québec, Canada; 4 Department of Education & Counselling Psychology, McGill University, Montréal, Québec, Canada

**Keywords:** feedback, attention resource capacity, learning, multiple object-tracking

## Abstract

Feedback is beneficial for learning. Nevertheless, it remains unclear whether (i) feedback draws attentional resources when integrated and (ii) the benefits of feedback for learning can be demonstrated using an attention-based task. We therefore (i) isolated feedback-specific load from task-specific load via individual differences in attention resource capacity and (ii) examined the effect of trial-by-trial feedback (i.e., present vs. absent) on learning a multiple object-tracking (MOT) paradigm. We chose MOT because it is a robust measure of attention resource capacity. In Study 1 participants tracked one (i.e., lowest attentional load condition) through four target items (i.e., highest load condition) among eight total items. One group (n = 32) received trial-by-trial feedback whereas the other group (n = 32) did not. The absence of feedback resulted in better MOT performance compared with the presence of feedback. Moreover, the difference in MOT capability between groups increased as the task-specific attentional load increased. These findings suggest that feedback integration requires attentional resources. Study 2 examined whether the absence (n = 19) or presence (n = 19) of feedback affects learning on the same MOT task across four testing days. When holding task-specific load constant, improvement in MOT was greater with feedback than without. Although this study is the first to isolate feedback-specific load in attention with MOT, more evidence is needed to demonstrate how the benefits of feedback translate to improvement on an attention-based task. These findings encourage future research to further explore the interaction between feedback, attention and learning.

## Introduction

Learning can be accelerated by providing additional information immediately after a task is completed (Wilbert, Grosche, & Gerdes, 2010). Feedback, defined as the presentation of task-relevant information to guide behavior, is considered a powerful tool for learning (Hattie & Timperley, 2007). However, one must attend to this additional information to use and integrate this knowledge in subsequent trials (Zeithamova & Maddox, 2007), and therefore the process of integrating feedback may require its own share of domain-specific cognitive resources (Maddox, Bohil, & Ing, 2004; Waldron & Ashby, 2001; Zeithamova & Maddox, 2006).

In general, successfully completing a task requires one to allocate sufficient cognitive resources to match the task's cognitive demands, and therefore learning is reliant on one's domain-specific resource capacity. A task's level of difficulty, cognitive demands, or the amount of cognitive resources required to successfully complete the task is referred to as the task's cognitive load (Paas & van Merriënboer, 1994; Sweller, 1988; Sweller, 1994). The successful completion of a task depends on whether one is able to match the task's cognitive load from their reserve of cognitive resources (Paas & Van Merriënboer, 1994). If feedback requires its own share of cognitive resources, successfully completing a task would rely on one's capacity to allocate resources to the feedback-specific load in addition to the task-specific load.

Previous research has suggested that feedback directly affects attention (Ashby, Alfonso-Reese, & Waldron, 1998; Ashby & O'Brien, 2005; Patalano, Smith, Jonides, & Koeppe, 2001; Wilbert et al., 2010). To adjust behavior to successfully complete the task at hand, feedback integration is broken down into three steps (Zeithamova & Maddox, 2007). First, the rule or procedure used is evaluated after an incorrect trial. Second, a decision is made whether to discard the procedure or establish a new rule altogether. Third, attention shifts from the old rule to a new method of completing the task. These three steps suggest the requirement of attention in order to integrate the information provided by feedback to change behavior for subsequent trials (Maddox et al., 2004; Waldron & Ashby, 2001; Zeithamova & Maddox, 2006).

Research examining the dynamic between feedback and attention has been limited to perceptual learning, memory-based studies, or both (Herzog & Fahle, 1998; Maddox et al., 2004; Waldron & Ashby, 2001; Zeithamova & Maddox, 2006). We propose that a multiple object-tracking (MOT) task could isolate feedback-specific load given the task's ability to characterize the allocation of attentional resources to task-specific load (Scholl, 2009) and individual differences in attention resource capacity (Tullo, Faubert & Bertone, 2018). MOT involves tracking relevant, target objects while ignoring physically indistinguishable, irrelevant, distractor objects as they move about a space over a short period of time. It is a robust measure of visual attention tapping into many of its subcomponents; selective, sustained, distributed and dynamic (Scholl, 2009).

Although most research in MOT is centered around understanding the mechanisms underlying visual tracking capability (Meyerhoff, Papenmeier, & Huff, 2017; Scholl, 2009; Suchow, Fougnie, Brady, & Alvarez, 2014), researchers have characterized MOT capability across developmental stages (Trick, Perl, & Sethi, 2005) and in typically and atypically developing populations (Brodeur, Trick, Flores, Marr, & Burack, 2013; Evers, de-Wit, der Hallen, Haesen, Steyaert, Noens, Wagemans, 2014; Koldewyn, Weigelt, Kanwisher, & Jiang, 2013). Most relevant to this study, research in MOT has isolated the specific load extraneous factors, such as short-term memory and working memory draw from one's attention (Alnæs, Sneve, Richard, Skåtun, Kaufmann, Nordvik, Andreassen, Endestad, Laeng, Westlye, 2015; Fougnie & Marois, 2006; Tombu & Seiffert, 2008). Therefore MOT may be an appropriate task to isolate and quantify feedback-specific load.

The benefits of feedback for learning have been well researched (Hattie & Timperley, 2007); however, the idea that feedback draws attentional resources has yet to be investigated. It is unclear whether successfully integrating the additive information provided via feedback depends on one's ability to allocate attentional resources to both task- and feedback-specific load. Addressing this question could demonstrate that the presence of feedback adds to the attention task's specific load, and consequently, affects performance.

We conducted two studies to examine whether trial-by-trial feedback draws its share of attentional resources from one's limited attention resource capacity as measured by MOT capability. We investigated this by manipulating the task-specific load in a MOT task and measuring performance with the presence or absence of feedback (Study 1). Moreover, we examined whether the inclusion of feedback would affect learning by assessing MOT capability across four testing sessions (Study 2). The results from these studies aim to examine the role of feedback in the allocation of attentional resources, and this role's interaction with learning.

## Study 1: Characterizing feedback-specific load

Identifying and characterizing feedback-specific load in an attention-based task can be made possible by manipulating the task's attentional load (Fougnie & Marois, 2006; Tombu & Seiffert, 2008). Research exploring MOT capability has demonstrated that manipulating task-specific load; specifically, the number of target items and speed, can help demonstrate the unique load any isolated factor holds (Alnæs et al., 2015; Fougnie & Marois, 2006; Tombu & Seiffert, 2008). This ability to characterize feedback-specific load is feasible because MOT can specifically and accurately target an individual's attention resource capacity (Alvarez & Cavanagh, 2004; Alvarez & Franconeri, 2007; Tullo, Faubert & Bertone, 2018).

Researchers have been able to isolate within-task factors that influence the allocation of attention during MOT task completion, including object set size (i.e., the number of target objects asked to track; Alvarez & Franconeri, 2007; Fougnie & Marois, 2006) and object velocity (Tombu & Seiffert, 2008). In addition to these within-task factors, MOT paradigms have demonstrated the effects of external factors on the allocation of attentional resources, including the role of (i) short-term memory, by tracking targets with unique identities (Makovski & Jiang, 2009; Oksama & Hyönä, 2016); and (ii) working memory, through the examination of MOT capability in combination with another task (Allen, Mcgeorge, Pearson, Milne, 2006; Fougnie & Marois, 2006; Zhang, Xuan, Fu, & Pylyshyn, 2010). Thus the attention-based MOT task is sensitive to the task-specific load manipulation and can be used to assess the unique load an external factor holds.

Study 1 assessed whether feedback draws attentional resources to be integrated for subsequent trials by measuring MOT capability, defined as the average speed participants could track all target items, and manipulating the task-specific load. Participants were asked to track one (i.e., lowest task-specific load condition), two, three, and four target objects (i.e., highest task-specific load condition) out of eight total objects. Feedback was presented on a trial-by-trial basis to half the participants. Speed was used as the outcome variable as it has been shown to be an accurate descriptor of attention resource capacity (Alvarez & Franconeri, 2007; Tombu & Seiffert, 2008; Tullo, Faubert & Bertone, 2018).

If the integration of feedback requires its share of attentional resources, then MOT capability (i.e., average speed scores) should be decreased (i.e., lower) for individuals that receive trial-by-trial feedback. Moreover, this difference between feedback conditions should be greater when task-specific load conditions are high because this requires more attentional resources from one's limited resource capacity compared to low levels of task-specific load. We hypothesize that in conditions of high task-specific load (i.e., tracking three and four target objects), the absence of feedback (i.e., the No Feedback condition) will result in greater MOT capability compared to conditions with the presence of trial-by-trial feedback (i.e., the Feedback condition). These findings would suggest that processing task-specific feedback would result in the allocation of a significant amount of attentional resources that is manifested under conditions where one's resource capacity is burdened by the high task-specific load (i.e., tracking three and four targets out of eight targets).

### Method

#### Participants

Sixty-four neurotypical adults (n_male_ = 35; n_female_ = 29) were recruited for this study (*M*_age_ = 23.88, *SD*_age_ = 3.03). To avoid any biases in attention, vision, or both, we excluded participants who (a) were taking medication for a pre-existing condition that would affect their attention (i.e., stimulants or sedatives), (b) had a diagnosis of attention deficit hyperactivity disorder (ADHD), (c) had a personal or family history of seizure disorders, and (d) had any condition that would affect their vision. Participants were equally and randomly assigned to either the Feedback or No Feedback condition. Table 1 represents a breakdown of age and scores on a standardized measure of intelligence and attention for all participants.

**Table 1.** Peripheral characteristics by condition in Study 1. *Notes.* Means and (Standard Deviations) are reported for age, intelligence, and attention. The Wechsler Abbreviated Scale of Intelligence—Second Edition (WASI-II) has an overall score comprised of both verbal and fluid reasoning intelligence subscale scores represented by the full-scale intelligence quotient (FSIQ). The Conners Continuous Performance Task—Third Edition (CPT-3) is a clinically validated measure of attention used in clinical settings to suggest deficits in attention. Attention (*d*’ *t-*score) is categorized as either above average: *t* < 45; average: *t* falls between and 54, below average: *t* falls between 55 and 59; and poor: *t* ≥ 60.

Condition	N	Sex: M/F	Age	FSIQ	CPT-3 d’ t-score
Feedback	32	16/16	24.31 (3.34)	109.27 (13.59)	49.75 (8.47)
No Feedback	32	19/13	23.44 (2.65)	104.77 (12.58)	47.84 (8.30)
**Total**	**64**	**35/29**	**23.88 (3.03)**	**107.02 (13.18)**	**48.79 (8.44)**

#### Measures

##### Multiple Object-Tracking (MOT)

**Figure 1.**
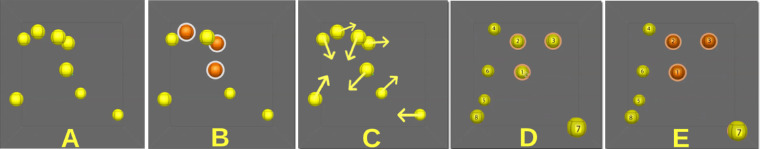
An illustration of the Multiple Object-Tracking task. These five stages represent one trial. (A) A presentation of all eight items (i.e., spheres) are displayed in the visual field. (B) The cued items are highlighted, allowing the participant to track these items. (C) The items move randomly throughout the visual field. (D) Numbers appear on all eight items, and the participant must identify the originally highlighted items. (E) Feedback is given to the participant; correct items are highlighted for those in the feedback condition only. Participants in the no-feedback condition did not have their answers verified. Instead the task restarted after (D).

A single MOT trial involved four key parts (Figure 1a-d). Trials began with a two-second presentation of eight physically indistinguishable items (i.e., spheres) positioned randomly throughout a three-dimensional (3D) visual field (Figure 1a). Dependent on the level of attentional load, either one, two, three, or four target items were indexed for three seconds by changing color from yellow to orange (i.e., four targets condition shown in Figure 1b), and participants were instructed to keep track of these cued items. The items returned to their original color (i.e., yellow) and moved randomly throughout the space (Figure 1c). In the visual tracking phase, there were instances where the items surpassed one another, collided with other items, or both. After eight seconds of movement, the items stopped, and numbers appeared to allow for the identification of the target items. To successfully complete the trial, participants had to identify all the target items, that is, those that were highlighted at the beginning of the trial (Figure 1d). Participants were instructed to fixate at a green dot in the center of the 3D space, and it was suggested that they use their peripheral vision to track the items throughout the movement phase.

The presentation of task-specific feedback was manipulated. Those in the Feedback condition were provided with information that either confirmed their correct response or highlighted the correct target items when there was an incorrect response (see Figure 1e). This information was displayed for 3 seconds. Participants in the No Feedback condition entered their responses and moved onto the next trial; there was no presentation of feedback (i.e., the information presented in Figure 1e was removed for the participants in this condition). There was no mention of the presence or absence of performance feedback. The research assistant administering the MOT task to the participant was instructed to avoid answering any questions about performance before, during, or in between trials. Instead, they explained to the participant that their performance could be discussed at the end of the study.

For both conditions, the speed of the items increased or decreased after each trial dependent on correct or incorrect responses, for each level of attentional load (i.e., one through four items; Faubert & Sidebottom, 2012). If the participant failed to identify the correct target items, subsequent trial speed decreased. Conversely, if the participant correctly identified all target items, subsequent trial speed increased. MOT capability was defined by the average speed at which participants successfully tracked all target items.

##### Wechsler abbreviated scale of intelligence—second edition

The Wechsler Abbreviated Scale of Intelligence—Second Edition (WASI-II) is a standardized measure of general intelligence for individuals between the ages of 6 and 89 years old. The intelligence quotient was generated as a Full-Scale Intelligence Quotient score (FSIQ) based on four subtests: Block Design, Matrix Reasoning, Vocabulary, and Similarities. The assessment time ranged between 30 to 45 minutes, which was dependent on the participant's performance.

##### Conners continuous performance task—third edition

The Conners Continuous Performance Task—3^rd^ edition (CPT-3) is a computer-based, standardized assessment of attention with age- and sex-based norms. Participants are asked to respond to letters of the alphabet that are flashed on the screen by pressing the spacebar as quickly and as accurately as possible for targets (i.e., any letter of the alphabet except for the letter “x”) and inhibiting a response when the nontarget (i.e., the letter “x”) appears. The task begins with a minute-long practice trial before the full 14-minute trial begins. Performance was defined by the task's primary variable *d’ t*-score: the ability to discriminate between target and non-target stimuli. The normalized *t*-score is based on age and sex. The CPT-3 categorizes performance as (i) above-average: *t*-score lower than 45; (ii) average: *t*-score between 45 and 54; (iii) below average: *t*-score between 55 and 59; (iv) poor: *t-*score greater than 60. The CPT-3 suggests that there is a presence of a deficit in attention if the participant has a *t*-score that is greater than 60 (i.e., poor performance; Conners, 2014).

#### Procedure

All participants completed the MOT task (i.e., all levels of load), the WASI-II, and the CPT-3, where the order of the tasks were randomized for each participant. For the MOT task, participants were asked to track one, two, three, and four out of eight items. The order for levels of load was randomized to control for fatigue. All participants were asked to track each level of load twice, for a total of eight separate blocks. Task speed was generated using a one up/one down staircase procedure (Kaernbach, 1991; Levitt, 2005). Initial speed was set at 68 cm/s displacement speed in virtual space and depending on the outcome on the previous trial, item speed increased or decreased by 0.05 log. Possible speeds ranged from 0.68 cm/s to 544 cm/s. The task ended once six inversions occurred. An inversion signified a shift from an incorrect to a correct answer and vice versa. After six inversions an average score was recorded (Legault, Troje, & Faubert, et al., 2012).

#### Design and analyses

To begin, we conducted preliminary analyses on peripheral characteristics to confirm that participants randomly assigned to either Feedback or No Feedback condition did not differ on either age, attention, or intelligence. Three separate between-subjects analysis of variance (ANOVAs) were conducted to explore whether these differences existed. Next, a multiple regression analysis was conducted to determine whether (i) feedback condition, (ii) load condition (log-transformed), and (iii) the interaction between load (log-transformed) and feedback condition predicted average speed score (log-transformed). This analysis was followed by four separate one-way between-subjects ANOVAs exploring the difference in MOT capability between the Feedback and No Feedback conditions.

### Results and discussion

Preliminary analyses were conducted on the standardized measures of attention and intelligence to explore whether there were any preconceived differences between participants randomly assigned to the Feedback or No Feedback condition. Specifically, conditions were matched on age: *F*(1, 62) = .1.31, *p* = 0.256, partial η^2^ = 0.02, 95% confidence interval (CI) [0, 0.14]; and on the clinically validated measure of attention (i.e., CPT-3 *d*’ *t*-score): *F*(1, 62) = 0.83, *p* = 0.365, partial η^2^ = 0.01, 95% CI [0, 0.12]. Moreover, four participants (two per condition) were non-English speakers; therefore they were unable to complete the Verbal Comprehension Index subtests. Nevertheless, conditions were matched on the Full-Scale Intelligence Quotient of the WASI-II, without the four non-English-speaking participants: *F*(1, 58) = 1.77, *p* = .188, partial η^2^ = 0.03, 95% CI [0, 0.16].

**Figure 2.**
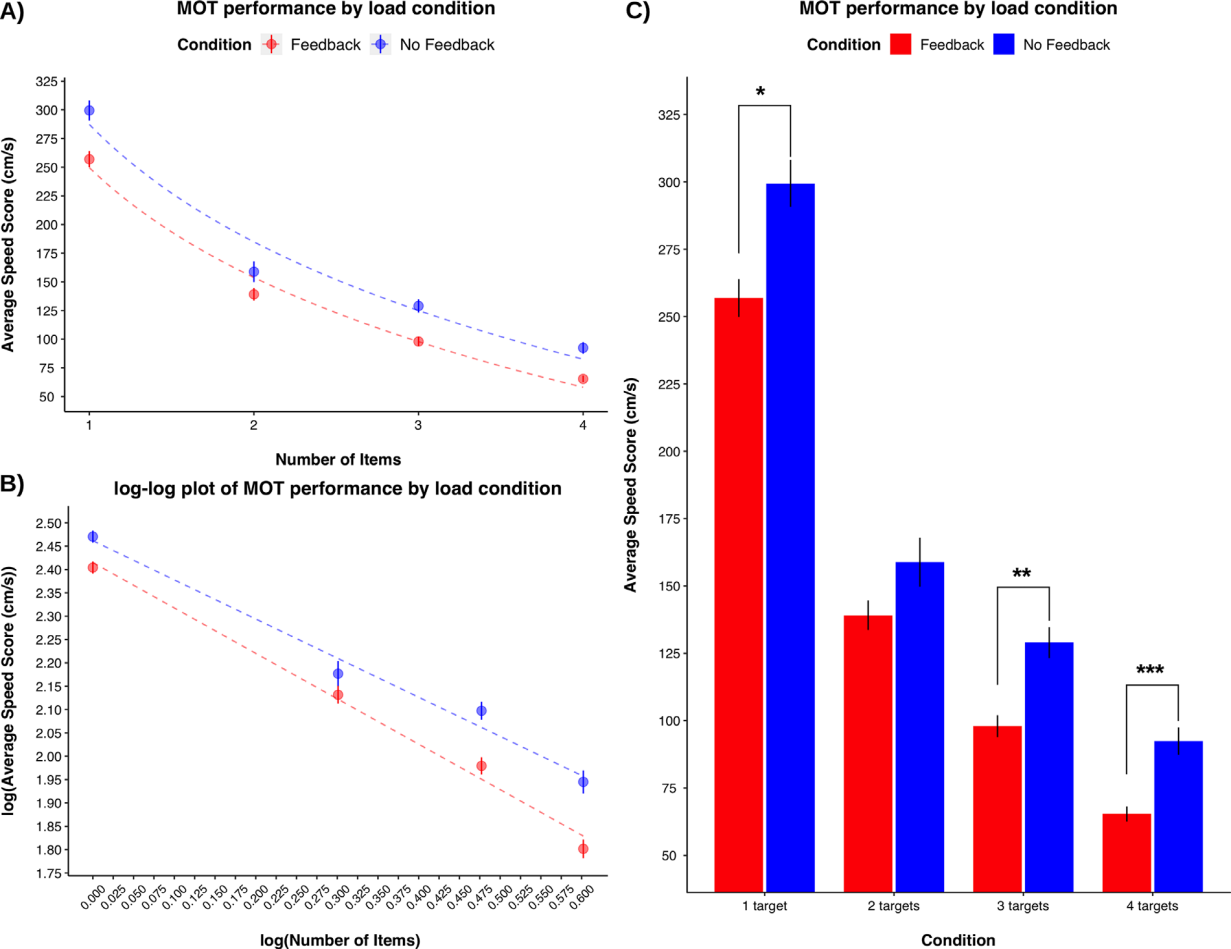
(A) The trend in MOT performance as load condition increases plotted (B) A log-log plot of MOT performance by load condition derived from Tullo, Faubert, and Bertone, (2018). (C) MOT capability across levels of attentional load (i.e., the number of target items asked to track) by Feedback and No Feedback conditions. * *p* < 0.001, partial η^2^ = 0.19; ** *p* < 0.001, partial η^2^ = 0.24; *** *p* < 0.001, partial η^2^ = 0.26.

Previous research has illustrated a decreasing logarithmic trend in average speed scores (i.e., MOT performance) as task load increases (i.e., increasing target items; Tullo, Faubert, & Bertone, 2018). Similarly, performance across both conditions matched the trend characterized by Tullo, Faubert, and Bertone (2018). Here, MOT performance decreased as a logarithmic function as task conditions (i.e., the number of target items increased; Figure 2a).

To compare these trends between conditions, we log transformed (i) the average speed score for MOT performance at each level of cognitive load and (ii) the number of target items tracked (i.e., load condition). With the log-transformed variables we conducted a regression analysis to examine whether (i) feedback condition, (ii) load condition, and (iii) the interaction between load and feedback condition predicted MOT performance (i.e., average speed score). The model was statistically detectable: *F*(3, 252) = 293.8, *p* < 0.001, *R*^2^ = 0.78, Adjusted *R*^2^ = 0.78. MOT's log-transformed load condition negatively predicted the average speed score: *b* = −0.97, *t*(252) = −21.94, *p* < 0.001; that is, average speed scores decreased as load condition increased on the log-log plot (Figure 2b). Feedback condition did not significantly predict average speed score: *b* = 0.05, *t*(252) = 1.81, *p* = 0.072, suggesting that the difference in the intercepts between the two feedback conditions were not statistically detectable. However, the interaction between load and feedback condition was a statistically detectable predictor: *b* = 0.13, *t*(252) = 2.15, *p* = 0.033, indicating that the rate of change or slope between the two feedback conditions differed.

The interaction between predictors demonstrated that the no-feedback condition had a greater slope than the feedback condition meaning that those that received feedback exhibited a greater decline in MOT capability as task-specific load increased compared to those that did not receive feedback. Thus we can interpret this difference in MOT capability across task conditions as feedback-specific load. This interpretation was confirmed by a direct comparison between feedback conditions at each level of load.

We conducted four separate one-way between-subjects ANOVAs to examine the effect of each level of load between feedback conditions. The results revealed a statistically detectable difference between Feedback and No Feedback conditions on MOT capability when tracking: one object: *F*(1, 62) = 14.48, *p* < 0.001, η^2^ = 0.19, 95% CI [0.04, 0.37]; three objects *F*(1, 62) = 19.43, *p* < 0.001, η^2^ = 0.24, 95% CI [0.07, 0.43]; and four objects *F*(1, 62) = 21.73, *p* < 0.001, η^2^ = .26, 95% CI [0.09, 0.45]. See Table 2 for means and standard deviations across levels of task-specific load. Figure 2c illustrates these differences in average speed scores across conditions. There was no statistically detectable difference in tracking two objects between conditions: *F*(1, 62) = 3.45, *p* = 0.068, η^2^ = 0.05, 95% CI [0, 0.20]; however, condition means trended toward significance. Additionally, we noted an increasing effect size as load increased. The difference in MOT capability between load conditions were more pronounced as load conditions increased from low (i.e., one and two targets) to high (i.e., three and four targets).

**Table 2.** MOT capability across levels of task-specific attentional load by feedback condition in Study 1. *Notes.* Means and (Standard Deviations) for MOT capability expressed as average speed scores (cms/s) across levels of task-specific load (i.e., tracking one, two, three, and four target items out of eight total items).

Condition	N	1 target	2 targets	3 targets	4 targets
Feedback	32	256.88 (39.73)	139.13 (30.85)	97.96 (22.93)	65.34 (15.97)
No Feedback	32	299.45 (49.27)	158.85 (51.55)	128.96 (32.52)	92.42 (28.72)

Previous research has been able to demonstrate that MOT paradigms are capable of highlighting the attentional load extraneous factors hold in attention by manipulating the MOT task's level of difficulty (Fougnie & Marois, 2006; Tombu & Seiffert, 2008). Our results demonstrated that feedback impacted tracking capability in both high (i.e., three and four target objects) and low task-specific load conditions (i.e., one target object, although results trended toward significance when tracking two target objects). This may be due to MOT's accuracy in defining the limits of attention resource capacity per level of attentional load (Alvarez & Franconeri, 2007; Tullo, Faubert & Bertone, 2018).

Defining MOT capability through the allocation of limited attentional resources has demonstrated that their distribution to MOT characteristics is a function of: (i) object set size, where attentional resources are distributed as: 1/*i,* where *i* is the number of target objects, and (ii) object velocity (Tullo, Faubert & Bertone, 2018). Tullo, Faubert, & Bertone (2018) identified individual differences in the limit of attention resource capacity at each level of load using the same outcome variable as presented here. Therefore the difference in average speed scores with the introduction of feedback in this study demonstrates that feedback possesses an attentional load, which exploits one's limited resource capacity. Moreover, these results illustrate that the effect of feedback-specific load increases as task conditions increase. Specifically, the differences in MOT capability between feedback conditions were more pronounced when tracking three and four target objects compared to one and two target objects. Thus these results suggest that attentional resources were divided between object set size, object velocity, and feedback. To our knowledge, this is the first study to demonstrate that feedback requires its share of attentional resources from one's limited attention resource capacity to be successfully integrated in future trials when completing an attention-based task.

## Study 2: Feedback and learning

The benefits of feedback are well documented in perceptual learning (Herzog & Fahle, 1997, 1998; Maddox, Bohil, et al., 2004; Shibata, Yamagishi, Ishii, & Kawato, 2009; Zeithamova & Maddox, 2006), memory-based (Maddox, Ashby, Ing, & Pickering, 2004), and language-based studies (Wilbert et al., 2010); but, to a lesser degree, attention-based studies. A study examining the effect of feedback on the ability to learn a fabricated language suggested that task-specific information is beneficial for learning (Wilbert et al., 2010). The authors discuss the importance of task-specific feedback given its role in directing attention toward relevant information. They hypothesize that providing information that is not relevant to task completion redirects attention towards irrelevant information. Although the authors conclude that task-specific feedback is ideal for learning, they could not conclude that any form of feedback drew no attention away from the task.

Attention, feedback, and learning mechanisms interact to facilitate task completion as well as improve performance. For instance, attentional resources are allocated to task features; but, learning mechanisms identify features that are relevant to successfully complete the task (Dosher, Han, & Lu, 2010; Sweller, 1988; Sweller, 1994). The dynamic between attention and learning mechanisms evolve into a cost-effective process by suppressing the allocation of attentional resources to irrelevant task characteristics and instead directing these resources to more appropriate task characteristics (Roelfsema, van Ooyen, & Watanabe, 2010). Moreover, the addition of corrective feedback helps the individual to discriminate between relevant and irrelevant task characteristics and this positively affects the rate of learning as demonstrated by significant improvements in task performance (Kelley & McLaughlin, 2012; Wolfe, Place, & Horowitz, 2007; Zeithamova & Maddox, 2007). The findings from Study 1 demonstrate that trial-by-trial feedback requires attentional resources in order to be processed, which was illustrated by decreased tracking capability. However, the language-based nature of the task used in Wilbert et al. (2010) suggests that they did not directly tap into the attention. Therefore, the impact feedback has on the interaction between attention and learning remains unclear.

To our knowledge, Wolfe et al. (2007) is the only other study examining the role of feedback and attention with MOT. Wolfe et al. (2007) examined whether feedback is beneficial for maintaining object identity throughout longer tracking duration (i.e., 10 minutes). By doing so this study demonstrated that the presence of feedback directs the allocation of attentional resources to relevant items; whereas the absence of feedback relies on the individual's attention resource capacity to maintain item identity (i.e., target or distractor) throughout the entire tracking duration. This study was the first to characterize feedback's role in redirecting attentional resources through the use of a MOT task. The results of Study 1 extend these findings by highlighting the attentional cost to incorporate these instructions to maintain or redirect attentional resources to target items. However, it remains uncertain whether the rate of learning on the same attention-based task differs between the absence or presence of task-specific feedback. Isolating the effect of feedback by examining the change in MOT performance across testing sessions can help characterize the interaction between attention, feedback and learning.

Study 2 examined whether the presentation of feedback was beneficial for learning on a MOT task. A difference in the learning rate between the two conditions would demonstrate that feedback impacts learning. Given the large body of research, which suggests that feedback is beneficial for learning, we hypothesize that those in the feedback condition would benefit from task-specific feedback. Specifically, the participants in the Feedback condition would have a greater change in MOT average speed scores from the baseline to the last testing day with all other factors being equal (i.e., age, performance on a separate, clinically validated measure of attention, and intelligence quotient (IQ)).

### Method

#### Participants

Forty neurotypical adults (n_male_ = 13; n_female_ = 27) were recruited for this study (*M*_age_ = 23.88, *SD*_age_ = 3.03). The same exclusion criteria were used for recruitment for Study 2. Table 3 represents participant characteristics for Study 2.

**Table 3.** Participant characteristics for baseline measures in Study 2. *Notes.* Means and (Standard Deviations) are reported. The WASI-II has an overall score represented by the full-scale intelligence quotient.

Condition	N	M/F	Age	FSIQ	CPT-3 *d’ t*-score
Feedback	20	6/14	23.45 (3.62)	112.09 (17.91)	48.36 (8.47)
No Feedback	20	7/13	23.3 (3.14)	112.67 (12.75)	49.22 (8.30)
Total	40	13/27	23.88 (3.03)	112.39 (15.07)	48.79 (8.44)

#### Measures

All measures used in Study 2 (i.e., MOT, CPT-3, and WASI-II) are the same as described in Study 1.

#### Procedure

All participants took part in four testing sessions over four consecutive days: baseline day 1 (D1), day 2 (D2), day 3 (D3), and day 4 (D4). The experiment was conducted over four days to assess the effect of learning the MOT task. During D1, a cognitive profile was defined for all participants based on measures of (i) general intelligence using the Wechsler Abbreviate Scale of Intelligence—Second Edition (WASI-II; (Wechsler, 2011) and (ii) attention using the Continuous Performance Test—Third Edition (Conners, 2014). After the cognitive assessment, a baseline measure of MOT capability was measured without the presence of feedback for all participants to assess MOT capability under the same circumstances for both experimental groups. This was done by removing the presentation of feedback, as illustrated in Figure 1 (E) and described in Study 1. For subsequent days (D2 to D4), participants were equally and randomly assigned to either (i) the group of participants receiving task-specific feedback immediately after each trial (n = 20) and (ii) the group receiving no feedback (n = 20). For baseline and D2 through D4, participants were asked to track four target items out of eight total items for a period of eight seconds. As was the case in Study 1, MOT capability was defined as the average speed the participant could track all target objects.

We controlled the presentation of trial-by-trial feedback similar to Study 1. Likewise, we defined tracking capability as the average speed the participant could track all target objects. In Study 2, we kept attentional load constant; participants were asked to track four target objects out of eight total objects for eight seconds to examine the effect of feedback on learning when conditions of attentional load were high. Once again, speed was used as our dependent variable to measure daily capability. Exclusive to the feedback condition, an average speed score was presented on the screen for three seconds at the end of the daily session. Participants in the no feedback condition were presented with an empty black screen for three seconds at the end of their daily session. Similar to Study 1, the research assistant administering the MOT task to the participant was instructed to avoid answering any questions about performance before, during or in between trials; and instead, they explained to the participant that their performance can be discussed at the end of the study.

#### Design and analyses

For Study 2, we conducted a one-way between-subjects ANOVA to examine whether there were any differences in baseline MOT capability between participants in the Feedback and No Feedback conditions. Next, an additional one-way between-subjects ANOVA was conducted to examine whether the standardized change in MOT capability from baseline to the last day of testing differed between participants in the Feedback and No Feedback conditions. We followed up this analysis with two paired samples *t*-tests (i.e., one per condition) to examine whether there was a significant improvement in MOT capability for each condition.

Furthermore, we conducted two multiple regression analyses to examine the learning trajectories across the two feedback conditions. Similar to the log-log plot in Study 1 and given that previous research has mapped learning trajectories in a typically developing adult sample on a logarithmic function (Faubert, 2013), we transformed the average speed score and the testing day by the natural log. Therefore the first model consisted of (i) testing day (log-transformed), (ii) feedback condition, and (iii) the interaction between testing day and condition predicting MOT performance (as defined by an average speed score). The second model, a hierarchical regression, examines the effect of feedback condition beyond baseline performance. The first step included baseline MOT performance predicting the standardized change in performance between baseline and D4. The second and third steps added feedback condition and the interaction between baseline and feedback condition, respectively.

We then examined the learning rate, defined as the standardized change in MOT capability between testing days to isolate when a change in performance occurred. A two-way mixed design ANOVA was conducted examining whether the rate of change in MOT capability differed across testing days (i.e., the within-subjects factor) and between Feedback conditions (i.e., the between-subjects factor). Follow-up one-way between-subjects ANOVAs were conducted to examine the difference in learning rates between conditions per testing day.

### Results and discussion

The data from two participants were removed from the analyses because they were deemed extreme outliers. One participant from each condition deviated more than 3 standard deviations from the mean in rate of change in average speed scores and were therefore removed from all analyses. The final number of participants used in the analyses was 38; 19 participants in the Feedback condition and 19 participants in the No Feedback condition.

**Figure 3.**
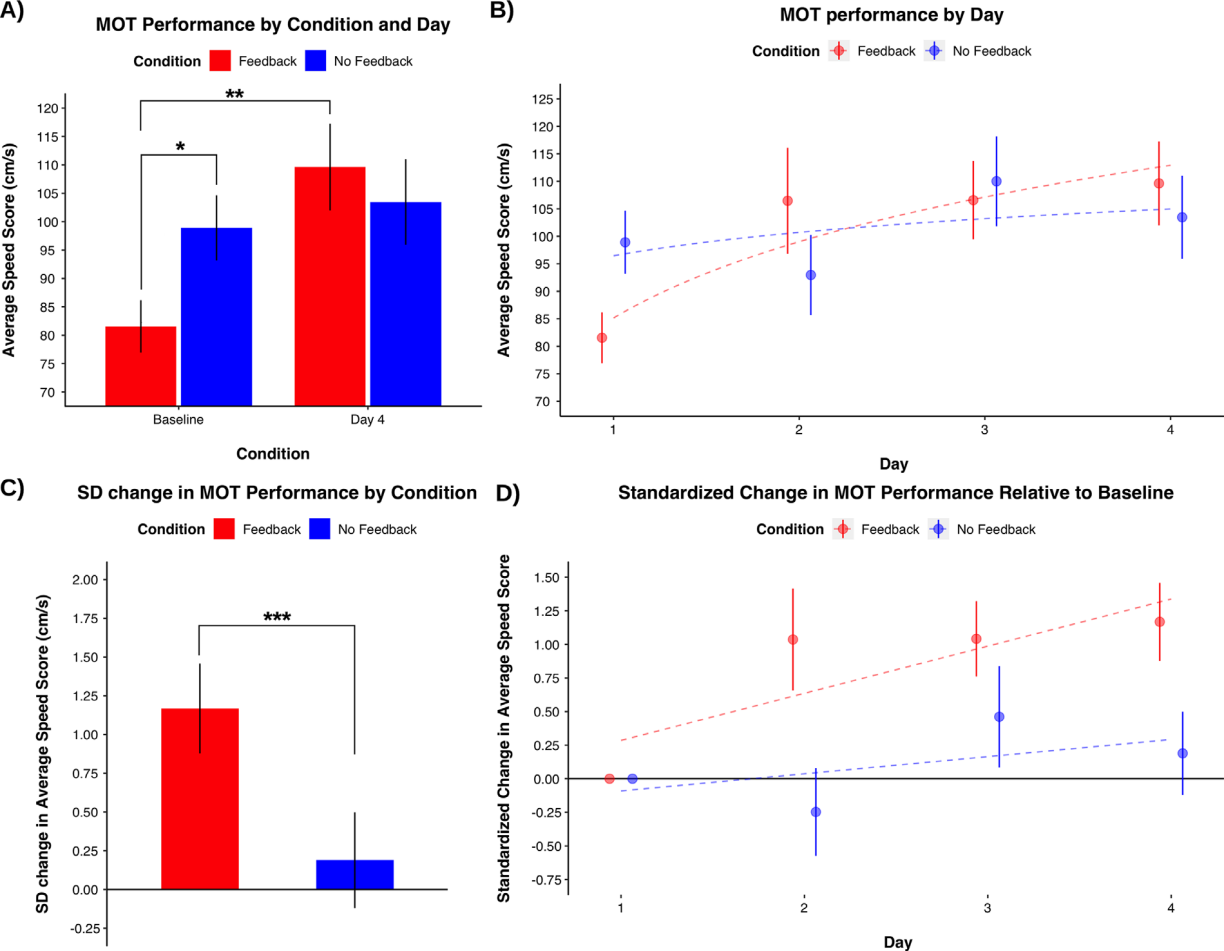
(A) MOT capability across all four testing days by feedback condition mapped onto learning curves. (B) The standardized change in MOT capability across four testing days by feedback condition. (C) MOT capability at baseline and day four by feedback condition. * *p* = 0.024 **p < 0.001. (D) The standardized change in MOT capability between feedback conditions. ***p = 0.027.

A one-way between-subjects ANOVA revealed that a difference in baseline MOT capability was statistically detectable between the two feedback conditions. Specifically, the group of participants that was assigned to the No Feedback condition could track targets faster (*M* = 98.92, *SD* = 25.01) compared to the group that was assigned to the feedback condition (*M* = 81.55, *SD* = 20.09): *F*(1, 36) = 5.57, *p* = 0.024, partial η^2^ = .13, 95% CI [0.00, 0.37] (Figure 3a).

To account for these a priori differences in baseline MOT capability, even though participants were randomly assigned to feedback conditions, we calculated the standardized change between baseline and D4. For instance, the standardized change consists of the difference between the new score and the baseline score, divided by the standard deviation of baseline scores for all participants: standardized change in MOT = (D4 MOT score − baseline MOT score) / standard deviation of MOT at baseline. A standardized change score considers the variance from the sample and can therefore account for an imbalance in baseline means between the two conditions. This descriptor is used throughout the field of cognitive training to examine improvement or change on cognitive measures (see Jaeggi, Buschkuehl, Jonides, & Perrig, 2008, for example).

We conducted a one-way between-subjects ANOVA using this metric to assess whether the presentation of feedback positively impacted learning the attention-based task. Our results revealed that the feedback group had a larger standardized change (*M* = 1.17, *SD* = 1.26) compared to the group that did not receive feedback (*M* = 0.19, *SD* = 1.35): *F*(1, 36) = 5.33, *p* = 0.027, partial η^2^ = .13, 95% CI [0.00, 0.36] (Figure 3b). A change of this magnitude suggests that those that received feedback improved by more than one full standard deviation, relative to the entire sample. Furthermore, the baseline means between conditions are separated by a z-score of 0.25. Therefore the change from baseline to D4 for the feedback group was more than four times larger than the distance between conditions at baseline.

Next, two paired samples *t-*tests (one per condition) were conducted to examine whether MOT capability improved for either condition. The results revealed that the those in the Feedback condition significantly improved from baseline (*M* = 81.55, *SD* = 20.09) to D4 (*M* = 109.63, *SD* = 33.26): *t*(18) = 4.03, *p* < 0.001, Cohen's *d* = 0.92, 95% CI [0.37, 1.46], whereas those in the No Feedback condition did not improve from baseline (*M* = 98.92, *SD* = 25.01) to D4 (*M* = 103.47, *SD* = 32.86): *t*(18) = 0.61, *p* = 0.549, Cohen's *d* = 0.14, 95% CI [−0.31, 0.59] (see Figure 3a). Notably, this study would not be the first to demonstrate an improvement in MOT performance with the inclusion of feedback.

Previous research has examined the change in MOT capability across multiple sessions (Faubert, 2013; Legault, Allard, & Faubert, 2013; Tullo, Guy, Faubert, & Bertone, 2018). Although the current study is the first to isolate the effect of feedback on MOT performance, the aforementioned studies demonstrated a learning effect for MOT with the inclusion of feedback. For instance, Tullo, Guy et al. (2018) examined the efficacy of MOT to improve performance on a separate, clinically validated measure of attention for children and adolescents diagnosed with neurodevelopmental conditions. This study demonstrated that repeated practice on the MOT paradigm improved performance by a standardized change of 0.62 (i.e., 41% increase as reported in the paper) after 15 sessions. The data from Tullo, Guy et al. (2018) suggests an improvement as early as four sessions. Moreover, Faubert (2013) demonstrated that the learning rates for MOT differed across levels of athleticism in a typically developed adult population. Similarly, the data from Faubert (2013) suggests a significant improvement after 15 sessions and as early as four sessions. Lastly, Legault et al. (2013) examined the differences in learning MOT between younger (i.e., ages 22–34) and older adults (i.e., ages 61–74). The study concluded that both groups significantly improved after five total training sessions. Therefore the contextualization of performance through the use of the standardized change score and the evidence of improvement on MOT performance with the presence of feedback from related work suggests that tracking capability can be improved or learned.

To compare the learning trajectories of the MOT task across the four testing sessions: (i) we mapped average speed scores onto a logarithmic curve, which has been characterized as a typical learning curve by previous research describing learning effects on a MOT task (Faubert, 2013; Tullo, Guy, et al., 2018) (ii) compared the trends using regression models. The feedback condition's trajectory followed this pattern by the equation: y = 20.019ln(x) + 85.152 at *R*^2^ = 0.85, whereas the group that did not receive feedback did not map onto this pattern as clearly: y = 6.1488ln(x) + 96.456 at *R*^2^ = 0.26. This discrepancy suggests that the MOT capability across a period of four days followed a pattern similar to a typical learning curve, whereas the group that did not receive feedback did not (Figure 3c).

To examine whether learning trajectories differed between feedback conditions, we conducted two multiple regression analyses. The first model, consisted of (i) the log of testing day (i.e., one through four), (ii) feedback condition, and (iii) the interaction between feedback condition and log of testing day predicting the log of the average speed score. However, this model was not statistically detectable: *F*(3, 148) = 2.61, *p* = 0.05, *R*^2^ = 0.05, Adjusted *R*^2^ = 0.03. Next, we conducted a hierarchical regression to examine the effect of feedback condition above and beyond baseline performance. The first step of regression model was statistically detectable: *F*(1, 36) = 6.10, *p* = .018, *R*^2^ = .14, Adjusted *R*^2^ = .12; where baseline threshold was a significant predictor of the standardized change in MOT performance from D4 to baseline: *b* = −0.02, *t*(36) = −2.47, *p* = 0.018. Adding condition as the next step resulted in a statistically detectable model: *F*(2, 35) = 4.39, *p* = 0.020, *R*^2^ = 0.20, Adjusted *R*^2^ = 0.16. However, the value added to the model with feedback condition was not statistically detectable: *F*(1, 35) = 3.14, *p* = 0.085, nor were the predictors baseline: *b* = −0.02, *t*(35) = −1.77, *p* = 0.085, and condition: *b* = −0.69, *t*(35) = −1.56, *p* = 0.127. The third and final step of the model examined whether (i) baseline speed scores, (ii) feedback condition, and (iii) the interaction between baseline scores and condition predicted the standardized change in MOT performance. This model was statistically detectable: *F*(3, 34) = 2.93, *p* = 0.047, *R*^2^ = 0.20, adjusted *R*^2^ = 0.14; nevertheless, the value added by the interaction predictor was not statistically significant: *F*(2, 34) = 5.40, *p* = 0.021, nor were there any statistically detectable predictors.

The results from the regression models demonstrate some inconsistency in the findings for Study 2. For instance, baseline tracking capability was associated with improvement in MOT performance. This finding coupled with the imbalance in tracking capability favoring the no feedback condition, suggests that more evidence is required to conclude that feedback improves performance on the attention-based task. Moreover, comparing the change in performance between testing sessions adds to the mixed findings.

We compared the rate of learning across daily sessions between the two conditions (see Figure 3d). We conducted a two-way mixed method ANOVA, where the standardized change interval of (i) D2 versus baseline, (ii) D3 versus baseline, and (iii) D4 versus baseline as the within-subjects factor and feedback condition as the between-subjects factor. The results of the ANOVA did not reveal a statistically detectable interaction between standardized change and condition *F*(2, 72) = 1.22, *p* = .301, partial η^2^ = .03, 95% CI [0, .13], nor a main effect of standardized change: *F*(2, 72) = 1.39, *p* = .255, partial η^2^ = .04, 95% CI [0, .14]; however, there was a between-subjects main effect of condition: *F*(1, 36) = 6.04, *p* = .019, partial η^2^ = .14, 95% CI [.00, .38]. Moreover, we conducted two separate post-hoc one-way ANOVAs examining the differences in standardized change intervals between conditions. Our results revealed that those receiving feedback had a greater standardized change between D2 and baseline: *F*(1, 36) = 6.60, *p* = .015, partial η^2^ = .15, 95% CI [.01, .39] and D4 and baseline (see above). There was no statistically detectable difference in the standardized change of D3 and baseline between conditions: *F*(1, 36) = 1.53, *p* = .225, partial η^2^ = .04, 95% CI [0, .23] (Figure 3d). Once again, these results support the notion that feedback is beneficial for learning on an attention-based task and that it took the group that did not receive feedback two testing days to achieve a statistically similar rate of change as the individuals that received feedback.

Taken together, these results, which indicate that exposure to feedback positively influenced learning on the MOT task, must be interpreted with caution, and this research question warrants further investigation. For example, future work is encouraged to explore the effect of this exposure by adding more testing sessions. Adding more than four training sessions would allow for an examination of the learning trajectories between conditions with higher-level statistical modelling. Overall, exploring the benefits of feedback in an attention-based task such as MOT would contribute to the knowledge that feedback aids performance from previous research examining this effect in perceptual-learning to language- and memory-based tasks (Ashby et al., 1998; Ashby & O'Brien, 2005; Herzog & Fahle, 1998, 1998; Maddox et al., 2004; Patalano et al., 2001; Roelfsema et al., 2010; Shibata et al., 2009; Waldron & Ashby, 2001; Wilbert et al., 2010; Zeithamova & Maddox, 2006). Therefore Study 2, which is the first to isolate feedback with repeated practice on an attention-based task, creates an avenue for future research to explain how attention is needed to consolidate the information provided by feedback.

Compared to Study 1, where there was no time to consolidate feedback between testing days, the 4-day learning period may have provided the participants with the opportunity to consolidate the feedback they were provided between daily sessions. The day long gap between testing can allow for the proper processing of Zeithamova and Maddox's (2006) steps for feedback integration. This is perhaps best evidenced by the sharp increase in capability once feedback is introduced to half the participants. Although the time between the presentation of feedback and the start of the next trial and/or testing session was not controlled for here, and given MOT's ability to highlight feedback-specific load, future research could explore what the optimal condition is for the presentation of feedback and the start of the next trial.

## General discussion and conclusion

The current study examined the role of feedback on attention and learning using a MOT task. Using a MOT paradigm, Study 1 demonstrated that feedback requires its share of attentional resources for the participant to process this additive information. Additionally, mixed results from Study 2 suggested that feedback may be beneficial for learning, as MOT capability improved after four daily sessions for individuals that received task-specific feedback; compared to no change by those that did not receive feedback. The current studies extend previous work by demonstrating that the incorporation of feedback can be influenced by the weight of the task itself (Hattie & Timperley, 2007), leaving feedback and task demands to compete for available attentional resources.

Although feedback competes against task demands for available resources, feedback can correct an erroneous allocation of attentional resources; and instead, reallocate these resources where needed. For instance, Wolfe et al. (2007) demonstrated that the information provided by feedback can be accessed within MOT trials to redirect resources to target items and suppress resources allocated to distractor items. The current study extends these findings by suggesting that feedback requires its share of attentional resources to manage this redirection of resources. However, the feedback manipulated in Study 1 did not isolate whether the individual (i) redirected resources to the target items or away from distractor items; or (ii) confirmed the item's identity mid-trial because the feedback provided on the proceeding trial has no relationship to the item's identity for the proceeding trial.

In Study 1 participants were exposed to feedback that provided information on their tracking capability. Specifically, the feedback provided to the participant confirmed or corrected their visual pursuit of target items. We can only speculate as to what this information would help guide in subsequent task performance. Perhaps it was a cue to allocate more attentional resources to instances of occlusion and collision. In fact, previous work has highlighted that these instances are where dropping and swapping commonly occur; and thus, maintaining object identification require additional resources (Iordanescu et al., 2009; Tinjust et al., 2008). A second hypothesis could be an alteration in tracking strategy. Although we advised participants to use their periphery to track target objects, the participants could have shifted their fixations between target items. These findings and subsequent hypotheses encourage future research to further delve into tracking strategies with the addition of eye-tracking data.

In Study 2 participants were exposed to the type feedback information as Study 1. We suggest that participants in Study 2's Feedback condition gained a significant advantage by adding feedback to the learning and attention dynamic. This was demonstrated by the significant increase in performance when feedback was introduced to participants in the Feedback condition. Feedback facilitates the reciprocal relationship between attention and learning by helping the individual target relevant features that need attentional resources (Kelley & McLaughlin, 2012; Wolfe et al., 2007; Zeithamova & Maddox, 2006). This allocation of attentional resources to task-relevant information (i.e., targets) allows for the conservation of resources that would otherwise be allocated to irrelevant task characteristics (Kelley & McLaughlin, 2012). The conservation of task-specific resources consequently decreases the task's cognitive load, resulting in an increased probability of a successful task completion and performance (Sweller, 1988). We therefore propose that feedback can be responsible for accelerating learning on the MOT task by increasing the participants’ ability to focus on relevant information (i.e., target objects) while ignoring irrelevant information (i.e., distractor objects).

Satisfying task demands with cognitive resources translates to successful task completion (Paas & Van Merriënboer, 1994). As a result, the ability to rapidly distinguish relevant from irrelevant information is another way in which feedback economizes the allocation of attentional resources to task demands (Sweller, 1994). This parallels the allocation of attentional resources in MOT (Alvarez & Cavanagh, 2004; Alvarez & Franconeri, 2007; Tullo et al., 2018). Both Study 1 and 2 defined MOT capability as the average speed in which the participant could successfully track all target objects (i.e., average speed scores). Feedback manipulation aside, MOT capability is contingent on both object velocity and the number of target objects. Individuals are unable to complete a trial when the combination of the task's load exceeds available resources (Alvarez & Franconeri, 2007; Tullo, Faubert & Bertone, 2018). Therefore the significant difference in average speed scores between conditions with the introduction of feedback highlights feedback-specific load. Additionally, as task load was manipulated by increasing the number of target items, the difference between task capability was more pronounced when feedback was introduced. This suggests that it becomes increasingly difficult to integrate feedback when task-specific load is high and expect successful task completion.

These results have identified feedback-specific load in MOT; however, more research is needed to characterize the magnitude of feedback-specific load. We advocate for future research to quantify the attentional demands of feedback by adding a secondary task to MOT and comparing performance to MOT with feedback. One example of this is Lapierre et al.'s (2017), where they flanked a MOT task with a separate visual working memory task. In the aforementioned study, the participant had to allocate a share of cognitive resources to store the information from the visual working memory task throughout the completion of the MOT task in order to successfully complete both tasks. Lapierre et al.'s (2017) study concluded that task disruption (i.e., decreased performance) depended on the order of the task's response phase rather than individual differences in cognitive capacity. Specifically, performance suffered if information about the preceding task had to be held throughout the completion of a separate task. Future research can borrow this methodology to quantify the attentional cost of feedback (i.e., defined by tracking capability) by comparing performance between two attention-based tasks completed simultaneously to performance on MOT with feedback.

There are implications on the finding that feedback requires additional attentional resources to guide learning that generalize beyond this specific area of research to education. These results imply that the additive cognitive demand of feedback should be taken into consideration: when designing a domain-specific cognitive task; and similarly, when designing a lesson plan. Moreover, there are distinct differences cognitive capacity and resource allocation between typically and atypically developing populations (Evers et al., 2014; Koldewyn et al., 2013; O'Hearn, Hoffman, & Landau, 2010; Van der Hallen, Evers, de-Wit, Steyaert, Noens, & Wagemans, 2015); therefore the addition of feedback-specific load needs to be considered in addition to the load imposed by a task or lesson plan, along with the individual's cognitive capability (Paas & van Merriënboer, 1994; Sweller, 1994).
